# Intra-Arterial Thrombolysis for Acute Central Retinal Artery Occlusion: A Systematic Review and Meta-Analysis

**DOI:** 10.3389/fneur.2018.00076

**Published:** 2018-02-21

**Authors:** Paul S. Page, Nicolas K. Khattar, Andrew C. White, Alexander C. Cambon, Guy N. Brock, Shesh N. Rai, Robert F. James

**Affiliations:** ^1^Department of Neurological Surgery, University of Louisville School of Medicine, Louisville, KY, United States; ^2^Department of Radiology, University of Louisville School of Medicine, Louisville, KY, United States; ^3^Department of Bioinformatics and Biostatistics, University of Louisville School of Public Health and Information Sciences, Louisville, KY, United States

**Keywords:** retinal artery occlusion, thrombolytic therapy, intra-arterial infusions, revascularization, meta analysis

## Abstract

**Background and purpose:**

Acute central retinal artery occlusion (CRAO) is a serious ophthalmologic emergency that may result in monocular blindness. To date, studies evaluating intra-arterial thrombolysis (IAT) have not shown a definitive clinical benefit. We have conducted a systematic review with a meta-analysis to effectively evaluate this treatment option.

**Methods:**

A systematic literature search was focused on studies containing five or more patients undergoing IAT that included a control group treated with standard therapy. Pooled meta-analysis was performed.

**Results:**

Five retrospective controlled studies and one randomized clinical trial were identified satisfying all inclusion criteria resulting in the analysis of 236 patients treated with IAT and 255 patients treated with ST. A pooled fixed effects analysis resulted in an estimated odds ratio of 2.52, 95% CI (1.69, 3.77) (*P* < 0.0001) favoring IAT.

**Conclusion:**

IAT is a promising therapeutic option for CRAO with great potential. Further randomized trials are needed to establish a significant benefit and ensure the safety of the intervention.

## Introduction

Acute non-arteritic central retinal artery occlusion (CRAO) is a rare ophthalmologic emergency. Less than 30% of CRAO patients will demonstrate a spontaneous improvement in visual acuity (VA) (1–3). Despite the severity of this condition, few effective therapeutic options exist. Current standard therapy (ST) includes a combination of non-invasive or minimally invasive therapies such as aspirin, topical beta-blockers, carbogen, ocular massage, and anterior chamber paracentesis. These therapies have all largely demonstrated limited efficacy, even though they remain the only therapeutic options commonly available (4, 5).

Intravenous and endovascular methods have been examined in CRAO. Intra-arterial thrombolysis (IAT) may reduce systemic side effects by potentially lowering the total dose needed compared to intravenous administration (6–8). Similarly, direct administration of thrombolytic agents to the target vessel may improve overall efficacy. This approach is an attractive therapeutic option given that systems of care that are currently being upgraded for the endovascular treatment of acute stroke *via* thrombectomy can be leveraged and utilized for rapid IAT treatment, despite the difference in the treatment paradigm (9).

The published results of IAT for CRAO have been inconsistent resulting in a controversial view of its efficacy. The most recent meta-analysis evaluating IAT for CRAO was conducted in 2000, reported on only 100 patients treated with IAT, and did not utilize any controlled studies (10). In this manuscript, we provide a systematic review of the most relevant literature and perform a meta-analysis of controlled studies evaluating the efficacy of IAT for improving VA in CRAO.

## Methods

This meta-analysis was performed in accordance with the ‘Preferred Reporting Items for Systematic Review and Meta-Analyses’ (PRISMA) (11). We conducted a literature search in MEDLINE and EMBASE for studies utilizing IAT in the treatment of CRAO from January 1, 1946 to January 1, 2015. Key words utilized included “retinal artery,” “intra-arterial fibrinolysis,” “intra-arterial thrombolysis,” and “intra-arterial thrombolytic.” Relevant articles were systematically reviewed for content and overall significance. Inclusion criteria for qualitative systematic reviews consisted of studies in English containing five or more patients with acute onset non-arteritic CRAO undergoing IAT with either urokinase or rt-PA. In order to be included for the quantitative meta-analysis, reports had to include a control group treated with ST.

The number of patients with visual improvement was extracted and odds ratios (OR) with 95% confidence intervals were calculated for each study. Visual improvement was defined as any improvement in VA in either the IAT or ST group. When more than one measure of visual improvement was reported, the reported primary outcome was chosen. When a primary outcome for visual improvement was not designated or apparent, the most restrictive definition of improvement was chosen.

The meta-analysis was then conducted using the inverse variance method for weighting studies and the DerSimonian-Laird estimator for quantifying heterogeneity. Both fixed- and random-effects model meta-analysis were performed and a forest plot was produced using Review Manager version 5.3.5. The initial analysis was exploratory and included only the retrospective non-randomized studies due to their presumed similar level of evidence. Our primary analysis was performed in identical fashion, but additionally included the results of the randomized trial.

SAS 9.3 was used to construct a mixed effects model to evaluate the effect of study quality on treatment outcomes and assess for suitability of including studies of different quality in the pooled meta-analysis. The mixed effect model utilized was conducted in accordance with the methods described in Sheu and Suzuki (12). This approach allowed modeling of the log odds ratio (log OR) as the response variable. The variance of the log OR for each study was derived from Argresti et al. (13).

The R package *meta* (R version 3.1.1, The R Foundation for Statistical Computing) was used to construct a funnel plot to test for publication bias of study outcomes with respect to study size, which included a formal test of funnel plot asymmetry. All studies included in the qualitative analysis were assessed for additional biases including selection bias. A summary of the qualitative analysis is provided in Table 1.

**Table 1:** Qualitative analysis of systematic review for intra-arterial thrombolysis treatment for central retinal artery occlusion.

	Type of study	No. of IAT subjects (total subjects)	Average time to thrombolysis in hours	Agent and dose	Study design features and major study limitations (bias)	Results
Mercier et al. (14)	Retrospective Case Series	14 (14)	8.0	t-PA	No control group, not included in quantitative meta analysis	6/14 (42.9%) had significant visual improvement correlating to improvement of ≥0.3 on the log MAR scale
				Mean: 35 ± 13 mg		

Ahn et al. (15)	Retrospective cohort study with control group	57 (101)	22.7	Urokinase	All patients (IAT and controls) met the same predetermined inclusion/exclusion criteria and were treated concurrently, which limited selection bias	Overall, 24/57 (42.1%) in the IAT group showed significant final visual improvement (improved log MAR ≥ 0.3) vs. 11/44 (25%) in the ST group (*P* = 0.09^b^; Ahn et al., Table 3)^c^
					Predetermined subgroup analysis for severity of CRAO (CRAO stages)	Patients with final visual outcome 20/200 (legally blind threshold) or better: 11/57 (19.3%) in IAT group vs. 2/44 (4.5%) in ST group (*P* = 0.026)
					Time from symptom onset to treatment significantly different between groups representing a potential selection bias that may favor IAT therapy	Of incomplete stage CRAO patients, 11/13 (84.6%) in the IAT group showed significant final visual improvement (improved log MAR ≥ 0.3) vs. 3/13 (23.1%) in the ST group (*P* = 0.002)
				Maximum: 500	The incomplete CRAO subgroup did not have significantly different time to treatment differences resulting in a relative decrease in selection bias within this subgroup	

Schumacher et al. (16)	Prospective randomized controlled trial	42 (82)	12.8	Maximum: 500	Prospective randomized controlled trial significantly reduces risk of selection bias. Planned enrollment 200 subjects (100 in each treatment group) Inclusion criteria included all non-arteritic CRAO patients regardless of CRAO stage (incomplete, subtotal, total) resulting in a heterogenous patient population. Relative number of incomplete stage CRAO is not known between groups and, therefore, it is possible there were different relative severity levels of CRAO between groups. This possibility could have influenced results. Randomization should reduce this risk, but performing stratified randomization with CRAO stage as one of the stratum may have reduced this allocation risk	A prespecified interim efficacy analysis of the available data on the first 70 enrolled subjects was performed. The probability to detect a significant difference between the two groups upon study completion (200 subjects enrolled) was calculated based on the data of the 70 patients while assuming that the remaining 130 patients would resemble the initial 70 patients. They calculated an 8.1% probability of a statistical difference in favor of the ST group and a 0.1% probability of a statistical difference in favor of IAT therapy. As a result, the DSMB recommended halting enrollment early. A total of 84 patients had been enrolled at this point with data available on 82 patients for final intention to treat analysis
						Overall, 24/42 (57.1%) in the IAT group showed significant visual improvement at 1 month (improved log MAR ≥ 0.3) vs. 24/40 (60%) in the ST group (*P* = 0.83)^c^
					Time to treatment was on average 2 h longer in the IAT group compared to the ST group, which could have resulted in a treatment bias unfavorable to the IAT group	Mean log MAR improvement of the IAT group was 0.447 (SD ± 0.545) and of the ST group was 0.443 (SD ± 0.549)

Zhang et al. (17)	Retrospective case series	49 (49)	<6	Urokinase	No control group, not included in quantitative meta analysis	At 6 months, 18/49 (36.7%) patients had regained VA to > 0.6
						35/49 (71%) had recanalization after IAT. Those with recanalization received greater recovery of visual acuity with a VA of 0.6 ± 0.04 in recanalized patients compared to 0.002 ± 0.0012 in non-recanalized patients (*P* < 0.05)
				200,000 IU to 1 million IU	Retrospective analysis of 49 consecutive patients treated by IAT within 6 h of presentation	16/21 (76.2%) had significant visual improvement within IAT group vs. 7/21 (33.3%) within ST group (*P* = 0.018) as defined by one line improvement on Snellen chart or one VA category improvement when VA is worse than 20/400^c^
				Mean: 626,000		
Aldrich et al. (18)	Retrospective cohort study with control group	21 (42)	9.3	t-PA	Consecutive CRAO patients enrolled but no consistent preddetermined inclusion/exclusion criteria, thereby representing risk for selection bias	
				Maximum: 20 mg	Time from symptom onset to treatment significantly different between groups representing a potential selection bias that may favor IAT therapy	7/21 (33.3%) had significant visual improvement within IAT group vs. 1/21 (4.8%) within ST group (*P* = 0018) as defined by 3-lines or more improvement on Snellen chart
				Multiple 3 mg aliquots delivered until VA improvement	Initial VA better for IAT group vs. ST group (Aldrich et al., Table 2) though not statistically different (*P* = 0.31) may still present a bias toward better outcomes in IAT group	
					Criteria for “significant visual improvement” in primary outcome easier to achieve compared to other studies (one line improvement on Snellen chart or one category improvement when VA worse than 20/400), although a *post hoc* analysis with a more restrictive 3-line improvement was also included	

Pettersen et al. (19)	Retrospective case series	8 (8)	9.7	t-PA	No control group, not included in quantitative meta analysis	8 cases of CRAO treated with IAT were identified but only 6 had VA follow-up in clinic. Of the two who did not have clinic follow-up, neither had improved 24 h after IAT. Therefore, 6/8 patients had documented improvement after IAT
				Range: 10—30 mg		6/6 patients with VA follow-up in clinic at least 1 month after IAT had VA improvement. 3/6 by 1 Snellen line and 3/6 improved by 2 or more Snellen lines

Arnold et al. (20)	Retrospective cohort study with control group	37 (56)	3.7	Urokinase	No significant difference in time from symptom onset to presentation (treatment) between the two groups. All presented in less than 6 hours as part of inclusion criteria minimizing selection bias	8/37 improved to a clinically significant ≥ 0.6 VA in the IAT group vs. 0/19 in ST group (*P* = 0.04)^c^
				Mean: 677,000 U	24 of 37 patients in the IAT group received anterior chamber paracentesis as one of the standard therapies while just 4 of 19 received this modality in the ST group (*P* = 0.004). This represents the only identified significant difference between groups (baseline characteristic or treatment), but does represent a potential source of treatment bias potentially favoring the IAT group	28/37 had any amount of log MAR improvement in the IAT group vs. 10/19 in the ST group (*P* = 0.13; derived from Arnold et al., Figures 1 and 2)

Butz et al. (21)	Retrospective case series	22 (22)	7.6	Urokinase	No control group, not included in quantitative meta analysis	7/22 marked improvement in VA, 2/22 slight improvement
				Mean: 642,000 SD 300 K		
				t-PA Mean: 27 ± 8 mg		

Schmidt et al. (22)	Retrospective cohort study with control group	62 (178)	10.8	Urokinase: 1 ml/min	No significant difference in time from symptom onset to treatment between the two groups (when comparing median times; *P* = 0.5), minimizing the potential for time to treatment selection bias	36/62 (58%) of patients in the IAT group demonstrated partial or distinct visual improvement vs. 34/116 (29.3%) in the ST group (*P* < 0.001)^c^
				200,000–1.3 million IU; Median: 1 million IU	Severity of CRAO Stage was statistically similar between groups. 10/62 (16.1%) patients were incomplete (best stage) vs. 29/116 (25%) in the ST group (*P* = 0.19). This would tend to favor visual improvement in the ST group and, therefore, this difference would not represent any bias in favor of the IAT group	In subgroup analysis (not pre-specified), only subtotal stage CRAO demonstrated significant visual improvement with IAT therapy. Partial or distinct improvement was seen in 24/47 (51%) in the IAT group vs. 15/83 (18.1%) in the ST group (*P* < 0.001)
				t-PA: 40–80 mg	Specific reasons why a given patient was treated with IAT vs. ST only were not given other than patients were allocated to the ST group when they had “contraindications” to IAT. This lack of specificity in the methods section raises a concern for selection bias	
				Median: 50 mg		
				IAT stopped once VA improvement identified		

Richard et al. (23)	Retrospective case series	53 (53)	14.0	t-PA	No control group, not included in quantitative meta analysis	35/53 improved at 3 months, no correlation with time
				Maximum: 40 mg		

Weber et al. (24)	Retrospective cohort study with control group	17 (32)	4.2	Urokinase	Patients had similar initial VA between groups	11/17 (64.7%) of patients had visual improvement in the IAT group compared to 5/15 (33.3%) in the ST group (*P* = 0.16)^c^
				100,000–900,000 IU	6 of 17 patients (35.3%) in the IAT group received anterior chamber paracentesis as one of the standard therapies while just 1 of 15 (6.7%) received this modality in the ST group (*P* = 0.09). While not a statistically significant difference between groups, this could represent a potential source of treatment bias potentially favoring the IAT group	
				Mean: 594,000	There was no predetermined specific inclusion/exclusion criteria and not all patients were treated concurrently, thereby introducing the risk of selection bias and treatment bias between groups	3/17 total recovery, 2/17 marked improvement, 6/17 slight improvement vs. 5/15 minimal improvement in ST
					Patients presented with similar times from symptom onset to treatment reducing time to treatment as a potential selection bias	

Schumacher and Schmidt (25)	Retrospective case series	35 (35)	4–2.5 days	Urokinase	No control group, not included in quantitative meta analysis	23/35 (66%) were either complete, marked, or definite but less marked improvement in VA. 10/35 (29%) total or marked improvement, 13/35 (37%) definite improvement
				Maximum: 1.2 million Units		
				t-PA		Continuation of prior studies (26–28)^a^
				Maximum: 70 mg		

*IAT, intra-arterial thrombolysis; t-PA, tissue plasminogen activator; LogMAR, logarithim of the minimum angle of resolution; CRAO, central retinal artery occlusion; ST, standard therapy; DSMB, data safety monitoring board; VA, visual acuity; IU, international units*.

*^a^Continuation of prior studies*.

*^b^Corrected P-value which differs from the study reported inaccurate P-value*.

*^c^Data used for pooled meta-analysis*.

## Results

### Systematic Review

**Figure 1:**
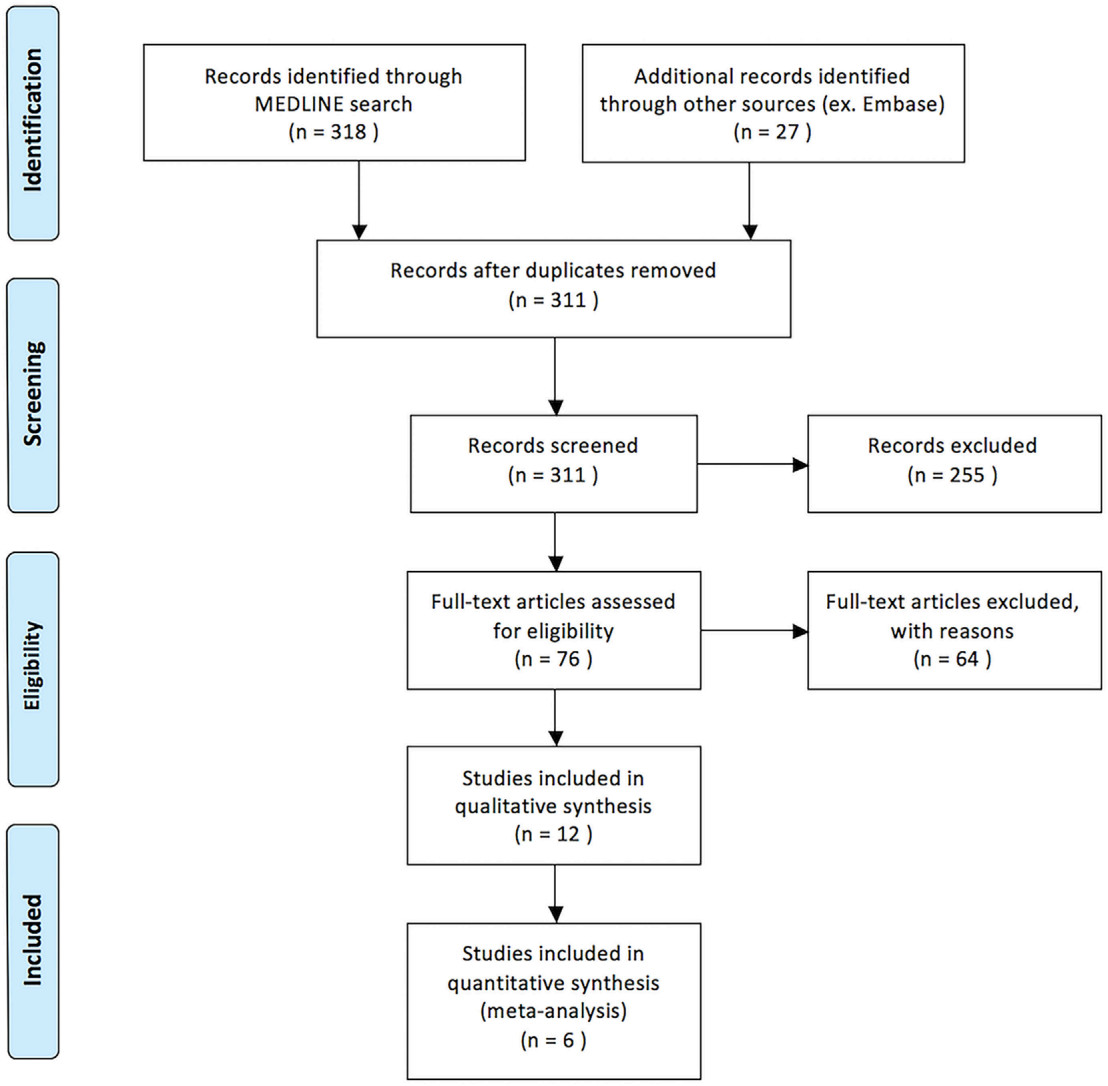
PRISMA flow diagram demonstrating method of systematic review.

Our search yielded 318 titles from MEDLINE and 27 titles from EMBASE (Figure 1). Seventy-six articles were identified as being relevant to our topic. Twenty-one relevant studies were included as they were containing five or more patients treated with IAT for acute non-arteritic CRAO (14–25, 29–34). Of these, nine were excluded either for not being written in English, representing duplicate data, or both. In total, 12 English language studies were identified as being relevant to the topic of discussion and satisfying all criteria for review. Eleven of these studies were retrospective in nature and one was a randomized controlled trial (14–25). A total of 417 patients treated with IAT are presented.

Upon review of the included studies, 236 of 417 patients (56.5%) treated with IAT demonstrated improvement in VA. Mean time from symptom onset to thrombolysis varied widely between studies ranging from 4.2 to 22.7 h. Mean age of patients treated with IAT was 61.9 years. Either urokinase or rt-PA was administered to patients in the IAT group. Urokinase dosing ranged between 200,000 and 1,300,000 IU. The maximum dose of rt-PA administered across all studies was 80 mg. Table 1 is a detailed summary of all 12 studies including their results and an assessment of bias.

### Meta-Analysis

Six studies fulfilled all inclusion criteria for evaluation in the quantitative meta-analysis. Overall, 236 patients were treated with IAT and 255 were managed conservatively with ST. Of those treated with IAT, 119/236 (50.4%) demonstrated an improvement in VA compared with 81/255 (31.8%) of those treated with ST (*P* < 0.005). Mean time from last known normal to treatment with IAT was 9.51 (range 1–172) h compared with 10.58 h for those treated with conservative treatment (range 2.0–22.5). No studies included demonstrated any statistically significant difference in age, gender, or presenting VA.

**Figure 2:**
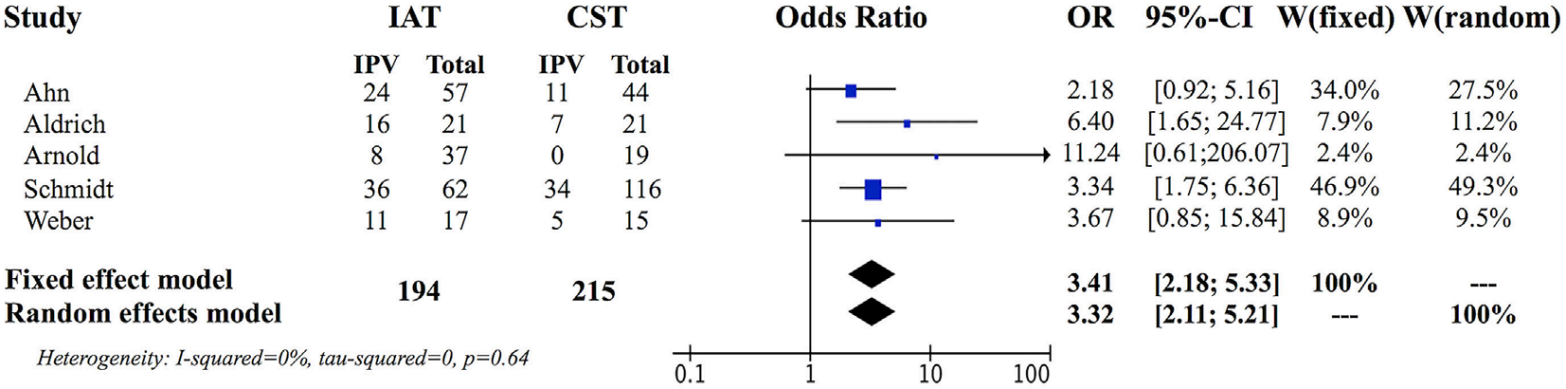
Forest plot evaluating five retrospective controlled studies.

The preliminary weighted pooled analysis included only the five retrospective non-randomized studies of a similar level of evidence (15, 18, 20, 22, 24). The estimated pooled ORs for fixed effects analysis was 3.41, 95% CI (2.18, 5.33), which was statistically significant (*P* < 0.0001) favoring IAT. The individual study ORs ranged from 2.18 to 11.24 (Figure 2) (15, 18).

**Figure 3:**
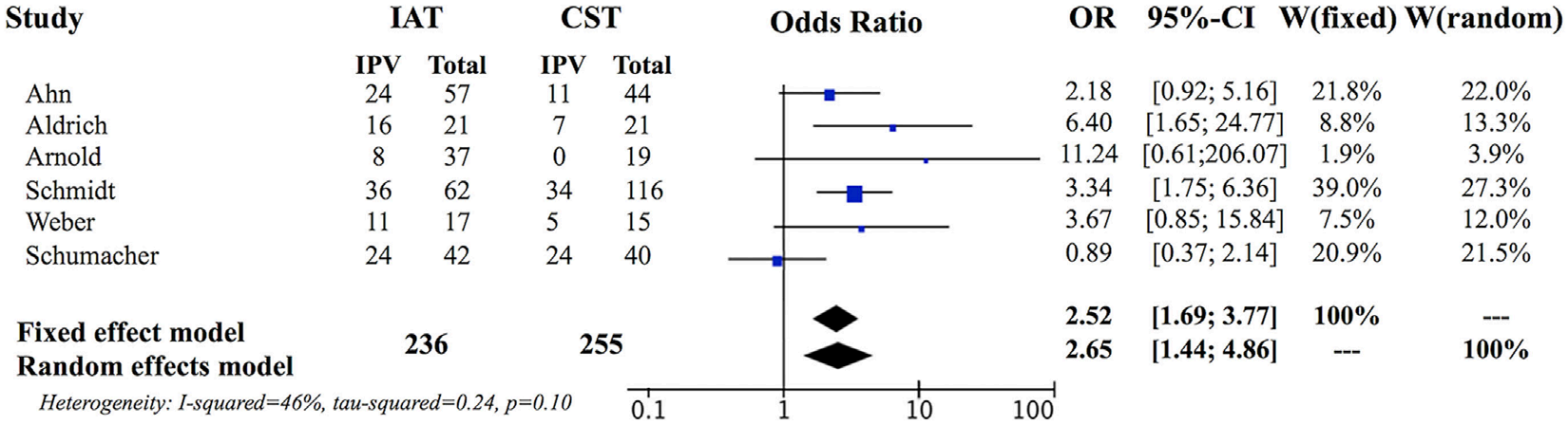
Forest plot evaluating five retrospective controlled studies and a randomized controlled trial. The addition of the randomized trial, which individually does not support IAT therapy, decreased the pooled odds ratio but it was still significantly in favor of intra-arterial therapy over standard therapy controls. There was an increase in heterogeneity, but this remained non-significant (*P* = 0.10) supporting the use of this method.

We then evaluated the randomized controlled trial by the European Assessment Group for Lysis in the Eye (EAGLE) equally with the retrospective studies (16). The individual OR for the EAGLE study was 0.89, 95% CI (0.37, 2.14) demonstrating no significant difference between IAT and ST in that study. Formal meta-analysis of all six studies, including the EAGLE study, resulted in fixed-effects pooled OR of 2.52, 95% CI (1.69, 3.77), which remained in favor of IAT (*P* < 0.0001) (Figure 3).

A mixed effects model comparing the treatment results of the EAGLE study compared to the retrospective studies showed a fixed effect difference for the estimated log OR that was −1.25 below the estimated log OR of the other five studies. This result did not represent a significant difference between the retrospective studies and the EAGLE study 95% CI (−2.84, 0.34), *P* = 0.087. Estimated variance of the random effect of study was <0.01 when the fixed effect was included. When the fixed effect was not included, the estimated variance of the random effect was 0.014.

**Figure 4:**
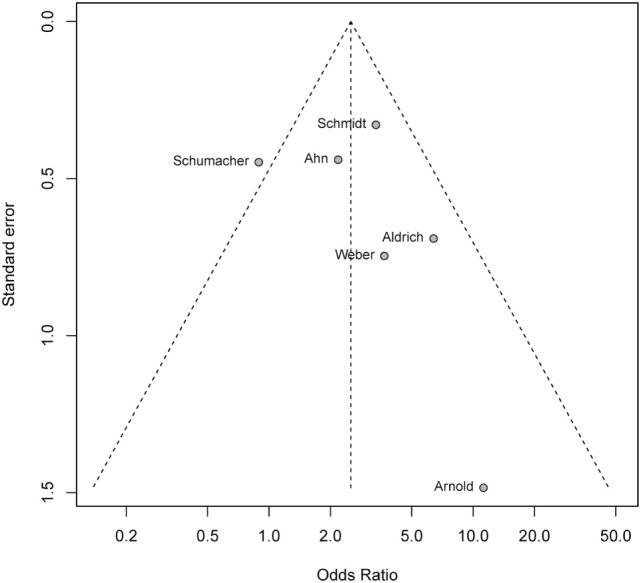
Funnel plot testing for publication bias. Formal test of funnel plot asymmetry with *P* = 0.47 showign a lack of publication bias.

Formal testing of funnel plot asymmetry was non-significant (*P* = 0.47) supporting a lack of publication bias of study outcomes with respect to study size (Figure 4).

Of the 236 patients examined, major complications included 4 groin hematomas, 2 intracranial hemorrhages, 5 transient ischemic events, 9 ischemic strokes (as documented on MRI imaging), and 1 hemianopia. Both intracranial hemorrhages were not associated with long-term disabilities with an mRS of 0 at the 1-month follow-up (16). Only two reported ischemic strokes were clinically significant and both resolved without permanent disability with an mRS of 0 at 2 weeks (15, 20). No studies reported long-term neurological deficit. Minor complications were more commonly reported including headache, tinnitus, and hyperesthesia.

## Discussion

While the use of IAT for CRAO is highly debated, studies directly comparing outcomes between ST and IAT generally favored the use of IAT. All five of the retrospective studies reported better visual outcomes for the IAT group compared to ST, yet, only 2 of those individual studies reached statistical significance in the chosen outcome measure where the lower end of the 95% confidence interval of the OR was greater than one (18, 22). Nevertheless, the OR for the retrospective studies significantly favored IAT therapy when the data was pooled for meta-analysis (Figure 2). Furthermore, the OR remained significantly favorable for IAT therapy, despite the addition of the EAGLE study, which does not support IAT therapy independently (16).

It is important to consider the varying primary outcomes and follow-up duration for studies included in the analysis. All studies reported the primary outcome for all studies [except for one which was not explicitly defined (24)] to be a change in best-corrected visual acuity (BCVA). In two studies, the primary outcome was determined at 1 month and at final follow-up, a clinically significant improvement being defined as logMAR ≥ 0.3 (18, 19). A single study defined clinically significant improvement as logMAR ≥ 0.6 (22) within 24 h after treatment, while two studies considered a positive primary outcome if there was one VA category improvement after intervention (21, 25).

Our meta-analysis supports the hypothesis that IAT significantly increases the likelihood of experiencing an improvement in VA compared with ST alone. Included studies showed dramatic variations in the efficacy of both IAT (23.5–80% in VA improvement) and conservative therapies alone (1–3, 29, 30).

### Assessment and Treatment of Bias within Included Studies

Qualitative analysis serves to address potential individual study design flaws and the potential for biases, specifically selection bias, within the included studies. The single most important driver of the positive results was the study by Schmidt et al. with the largest sample size and a significant treatment effect favoring IAT (22). It also had a favorable assessment of bias with excellent matching of time from symptom onset to time of treatment in both groups and likewise excellent matching of the stage of CRAO occlusion between groups.

Control patients were not always treated concurrently in the various studies. Determination of treatment with ST in some patients depended on the fact that the medical center was not yet performing IAT for CRAO at that time point. *A priori* application of inclusion and exclusion criteria was not consistently applied to any of the retrospective studies except for Ahn et al. introducing additional risk for selection bias (15). There were no differences in baseline characteristics except in the time from symptom onset to initiation of treatment parameter, which tended to be longer in control groups of retrospective studies potentially favoring IAT.

The variation in therapeutic regimens including type of treatment, dose, duration, and timing varied between patients within each study and between the studies in both IAT and ST groups introducing the potential for treatment bias. All the retrospective studies included in the analysis had considerable variability in both the ST and IAT treatment regimens.

The choice of outcome measure to assess the effectiveness of CRAO therapy varied considerably between studies. Some studies used descriptive terminology for assessments of BCVA improvement, while others used a quantitative analysis involving LogMAR scores derived from BCVA improvement. Likewise, there was significant variation in the definition of a “good outcome” for treatment, ranging from any visual improvement to significant quantified visual score improvement. The timing of follow-up was also inconsistent as some studies evaluated immediate outcomes, while others focused on delayed outcome measures ranging from 1 month to 1 year or more.

### Inclusion of Randomized and Non-Randomized Studies in the Pooled Analysis

Different approaches were considered for sensitivity analysis to account for differences in study quality. One strategy involves assigning higher weight to studies with a level of evidence. Most weighting methods are considered arbitrary and study quality should not modify the precision of the pooled estimates (35). An alternative approach focuses the sensitivity analysis on the components of study quality considered most important to the meta-analysis. We performed a mixed effects approach utilizing a fixed covariate to identify the overall “component of study quality,” which we considered important (35, 36). Our study utilized an indicator variable and separated the individual studies into two categories: those that are prospective randomized trials and those that are not. A significant result of this fixed effect covariate would indicate that the EAGLE study has a significant difference in treatment effect compared to the other studies. We used a mixed effects model analysis, which resulted in −1.25 on the log OR scale for the EAGLE study lower than the non-randomized studies. The difference was not statistically significant and, therefore, our analysis of the impact of study quality shows no significant difference in overall treatment effect between studies of different quality. This supports the decision to pool all controlled studies meeting inclusion criteria regardless of level of evidence.

Even though the EAGLE study was a randomized controlled trial, significant concerns have been raised from various experts regarding its study design and its inclusion and exclusion criteria (37–39). We used the inverse variance method was used to calculate OR and *P*-value and obtained similar results using the Mantel–Haenszel method and the random effects model. The test of heterogeneity was not statistically significant (*P* = 0.1) and supported the models used.

### Clinical Relevance

Despite the positive outcomes of this meta-analysis, we do not recommend universal acceptance of IAT for CRAO. The inconsistent results that we observed based on the lack of efficacy for IAT seen in the only randomized trial and the divergence of these results from prior retrospective studies raises important questions.

Study design plays an important role in evaluating an intervention and significantly affects the likelihood of observing the desired effect. Given the possible flaws seen in the EAGLE trial, efforts should be directed at identifying study design choices of the EAGLE study that may have prevented seeing a positive treatment effect. The inclusion criteria of the EAGLE trial were broad and resulted in a heterogeneous study sample with regard to initial severity of CRAO. CRAO is classified into stages, which include incomplete, subtotal, and total subtypes in the order of increasing severity. Evaluation of the degree of arterial occlusion is based on fundoscopic findings or angiography and has been shown to correlate with visual outcomes after treatment with thrombolytics. Clinically significant improvement in VA was most likely to be seen in cases of incomplete CRAO (50–76.9%) as opposed to subtotal (10.6–14.3%) and total occlusion (0%) (15). A potentially effective strategy for future randomized trials may be to enroll CRAO patients who are in the incomplete stage, the subtotal stage or a combination of both stages. This strategy would exclude the total stage where an IAT treatment effect is not likely to be seen. This would serve to increase average treatment effect, decrease variance, and thereby decrease the sample size needed for future studies.

Time from symptom onset to fibrinolysis is another significant factor in the treatment of CRAO. Current results have inconsistently demonstrated an association between time to treatment and functional outcomes (39–41). While improvements can be observed in patients treated with IAT well after 6 h, we speculate the effectiveness of the intervention to lessen as time to treatment lengthens, in a similar fashion to endovascular stroke therapy (37, 38, 42). Based on prior evidence, multiple time points for intervention should be considered (41, 43).

Fear of serious complications associated with IAT is frequently cited, yet, our review did not demonstrate a heightened risk for long-term neurological complications.

### Limitations

While our meta-analysis was able to include a large number of patients, significant limitations are present. One limitation is the inherent treatment selection bias found in non-randomized studies, which can lead to confounders being over-represented in a given cohort. The decision on whether or not to perform IAT was sometimes related to time from symptom onset. Increased time from visual change to therapy in only one group would be expected to produce worse outcomes thus skewing results. An additional limitation is that our study did not evaluate the degree of visual improvement. In future trials, stratification of visual improvement would help quantify the effectiveness of the intervention and would help better confirm the optimal timing of the intervention (44). Another limitation was the inclusion of studies of varying levels of quality of evidence. While it is reasonable to include studies of different quality in a meta-analysis, it is not ideal. An additional important limitation is our own bias approaching this topic. Additional non-biased studies are needed to objectively assess the merit of this intervention.

## Conclusion

The EAGLE study is the only randomized trial and, considered in isolation, does not support IAT therapy. However, lessons from the acute stroke trials reinforce that it would be inappropriate to dismiss a promising treatment for CRAO based on a single randomized trial. This meta-analysis evaluating all controlled studies reporting IAT therapy for CRAO demonstrated a pooled OR significantly favoring IAT treatment. Our results support that further investigation with additional well-designed randomized controlled trials is necessary prior to establishing universal recommendations about IAT.

## Author Contributions

Conception, approved the final version on behalf of all authors, and study supervision: RJ. Design: RJ, PP, AC, and SR. Acquisition of data: PP, NK, AW, and AC. Analysis and interpretation of data: all authors. Drafting the article: RJ, PP, NK, AW, AC, and GB. Reviewed submitted version: all authors.

## Conflict of Interest Statement

The authors declare that the research was conducted in the absence of any commercial or financial relationships that could be construed as a potential conflict of interest.
